# Trapeziectomy and Mini TightRope Suspensionplasty for First Carpometacarpal Joint Arthritis

**DOI:** 10.7759/cureus.67695

**Published:** 2024-08-24

**Authors:** Tapan Das, Jitendra Mishra, Shivam Chawla, Nego Zion

**Affiliations:** 1 Department of Orthopaedics, Institute of Medical Sciences and SUM Hospital, Bhubaneswar, IND

**Keywords:** suspensionplasty, trapeziectomy, arthritis, cmc, mini tightrope

## Abstract

Introduction: First carpometacarpal (CMC) joint arthritis is a common debilitating condition affecting thumb function. Surgical management often involves trapeziectomy to alleviate pain and restore functionality. The suspensionplasty techniques maintain the trapezial height after trapeziectomy. The older techniques used the help of ligamentoplasty by taking the flexor carpi radialis or the abductor pollicis longus. A new technique of suspensionplasty where the first metacarpal is suspended to the second by means of a strong suture material (fiberwire) and tied with help of a suture button (Mini TightRope; Arthrex, Naples, FL, USA). This technique is less invasive than the previous ligamentoplasties, because there is no need to harvest another nearby tendon. The addition of Mini TightRope suspensioplasty aims to stabilize the joint, potentially improving outcomes.

Methods: A retrospective analysis was conducted on 20 patients undergoing trapeziectomy and Mini TightRope suspensioplasty between January 2022 and December 2023. Preoperative and postoperative assessments included pain scores, grip strength measurements, range of movement evaluations, and patient-reported outcomes using standardized questionnaires.

Results: Significant improvements were observed postoperatively in pain relief, with Visual Analog Scale scores decreasing from 7.8 preoperatively to 1.2 at six months follow-up. Grip strength increased by an average of 35%, and 85% of patients achieved near-normal range of movement. Patient-reported outcomes indicated high satisfaction rates, with enhanced ability to perform daily activities.

Conclusion: Trapeziectomy combined with Mini TightRope suspensionplasty demonstrates favorable outcomes in managing first CMC joint arthritis. This approach effectively reduces pain, improves grip strength, and enhances functional capabilities, underscoring its role as a promising surgical option for patients seeking relief from thumb arthritis.

## Introduction

First carpometacarpal (CMC) joint arthritis, primarily affecting the thumb, is a prevalent condition causing significant pain and functional impairment in affected individuals. This degenerative disease typically arises from wear and tear of the joint's articular surfaces, leading to inflammation, stiffness, and reduced range of motion. Patients often experience difficulties in performing everyday activities such as gripping, pinching, and grasping. Surgical intervention becomes necessary when conservative measures fail to provide adequate relief. Among the surgical techniques employed, trapeziectomy has been widely accepted as a standard procedure for alleviating pain by removing the arthritic trapezium bone [1]. However, concerns regarding instability and loss of thumb function post trapeziectomy have prompted the development of adjunctive procedures aimed at maintaining joint stability while preserving thumb mobility. Several surgical techniques have been described with no one superior to the other [2]. One such innovative technique is the combination of trapeziectomy with Mini TightRope (Arthrex, Naples, FL, USA) suspensionplasty. This approach not only addresses the structural deformity but also aims to stabilize the first CMC joint through a minimally invasive method and allow accelerated rehabilitation [3-5]. By securing the thumb metacarpal to the remaining carpal bones with a Mini TightRope device, joint stability is theoretically enhanced, potentially optimizing functional outcomes and reducing the risk of postoperative complications. This paper aims to explore the functional outcomes and efficacy of trapeziectomy combined with Mini TightRope suspensionplasty in patients with first CMC joint arthritis. Through a prospective analysis of patient data and clinical outcomes, we seek to evaluate the impact of this combined surgical approach on pain relief, grip strength, range of motion, and overall patient satisfaction. Additionally, we aim to discuss the theoretical advantages of this technique in preserving thumb function and improving quality of life for individuals suffering from thumb arthritis. By critically assessing the existing literature and presenting our findings, we aim to contribute to the evolving understanding of surgical options for first CMC joint arthritis and provide insights into optimizing treatment strategies to better meet the needs of affected patients.

## Materials and methods

**Figure 1:**
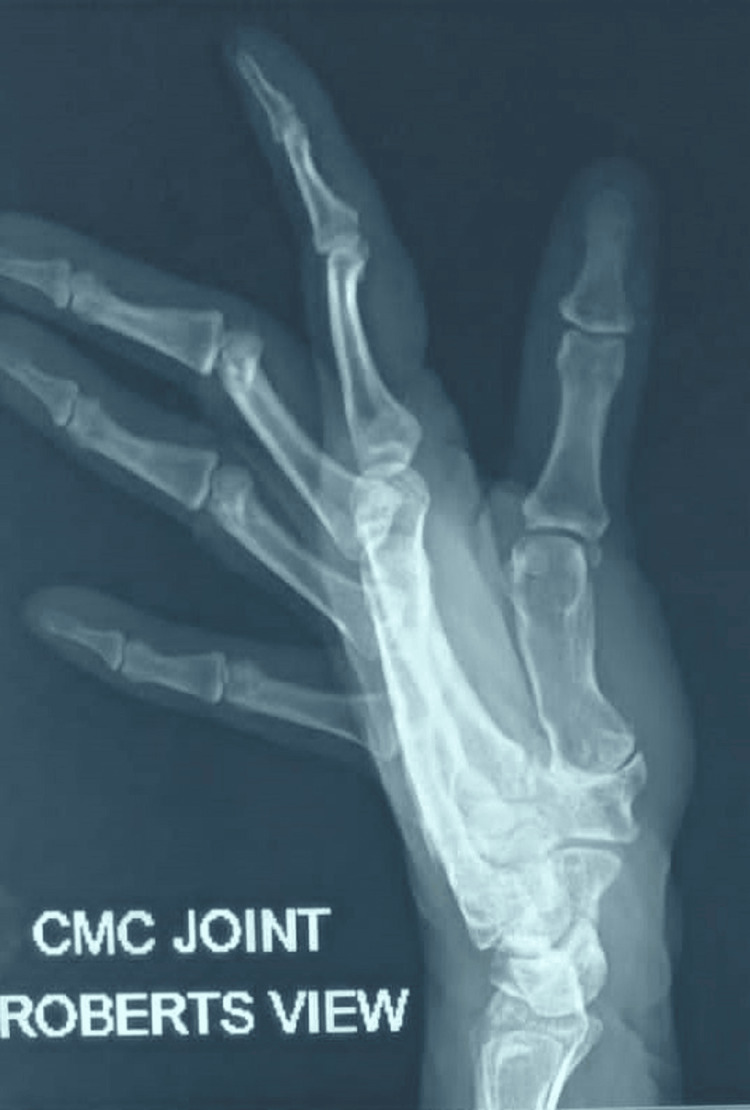
CMC Joint Roberts View

**Figure 2:**
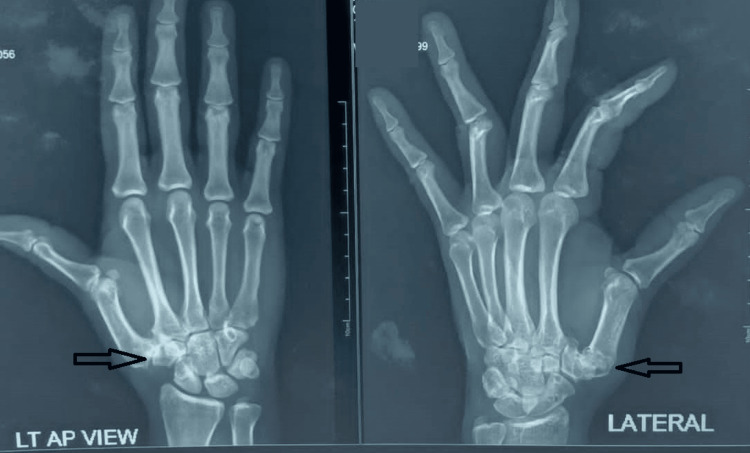
CMC Joint Anterior-Posterior (AP) and Lateral Views

We obtained approval through the institutional review board of the Institute of Medical Sciences and Sum Hospital (IEC/IMS.SH/SOA/2022/247A). All patients who underwent trapeziectomy and suspensionplasty and under a single surgeon's care with Current Procedural Terminology code 25447 met the inclusion criteria. All patients had been unsuccessful with conservative therapy, which included nonsteroidal anti-inflammatory medications, orthotic placement, and/or corticosteroids injection. Preexisting regional trauma or damage, further reconstructive surgery for non-CMC arthritis, and significant unrelated comorbidities were among the exclusion criteria. Records that satisfied the inclusion criteria were examined for radiographic anomalies such as scaphotrapezial abutment or device failure, as well as for the existence of ongoing discomfort or dysfunction and subsequent surgical operations. Depending on how they were created, problems were classified as either non-implant-related or implant-related. A single-centre prospective cohort study was conducted from January 2022 to December 2023 at the Department of Orthopaedics at the Institute of Medical Sciences and Sum Hospital, Bhubaneshwar, India, focussing on adults with first CMC joint arthritis. Twenty eligible patients underwent treatment with trapeziectomy and suspensionplasty with Mini TightRope fixation during the study period. Data collection included patient demographics, preoperative X-rays (Figures 1, 2), comorbidities, surgery duration, fluoroscopic imaging and comparison of post-operative pain, grip strength, range of movement and functional assessment (Table 1). The mean and standard deviation were used to describe the continuous variables and frequencies and percentages for the categorical variables.

**Table 1:** Demographics of operated patients before surgery a Visual Analouge Pain Score 1-10 b Preoperative Quick Disabilities of the Arm, Shoulder and Hand questionnaire

n	20
Age, median (range)	53.5(38-68)
Gender (male/female)	5/15
Pain (vas^a^ score)	8.3(7-9)
Q-dash^b^ , median (range)	59.4

Surgical technique

The patients were operated under tourniquet control and infraclavicular block in spine position. The procedure was done by the same head surgeon with a team of other surgeons. A 3-4 cm dorsoradial skin incision was made over the trapeziometacarpal joint. Capsule was incised and full trapeziectomy was done and wash was given and any residual bone fragments if present were removed. A separate 2 cm incision was given between the second and third metacarpal bases. The ulnar base of the index metacarpal was visualized which is the eventual exit point of the K-wire. A 1.1 mm tapered suture passing K-wire, starting on the proximal dorsoradial aspect of the first metacarpal and as close to the base as possible was given towards the second metacarpal base. The trajectory is to place the K-wire within the proximal one-third of the second metacarpal. While giving the K-wire, hand should be relaxed and in neutral position. The position was confirmed with fluoroscopy. The Mini TightRope was passed using the K-wire and tightening was done. Overtightening is not recommended as it can lead to decreased range of motion. Range of motion can be checked under fluoroscopy and further knots can be given (Video 1).

**Video 1:** Surgical Technique

Postoperative care

**Figure 3:**
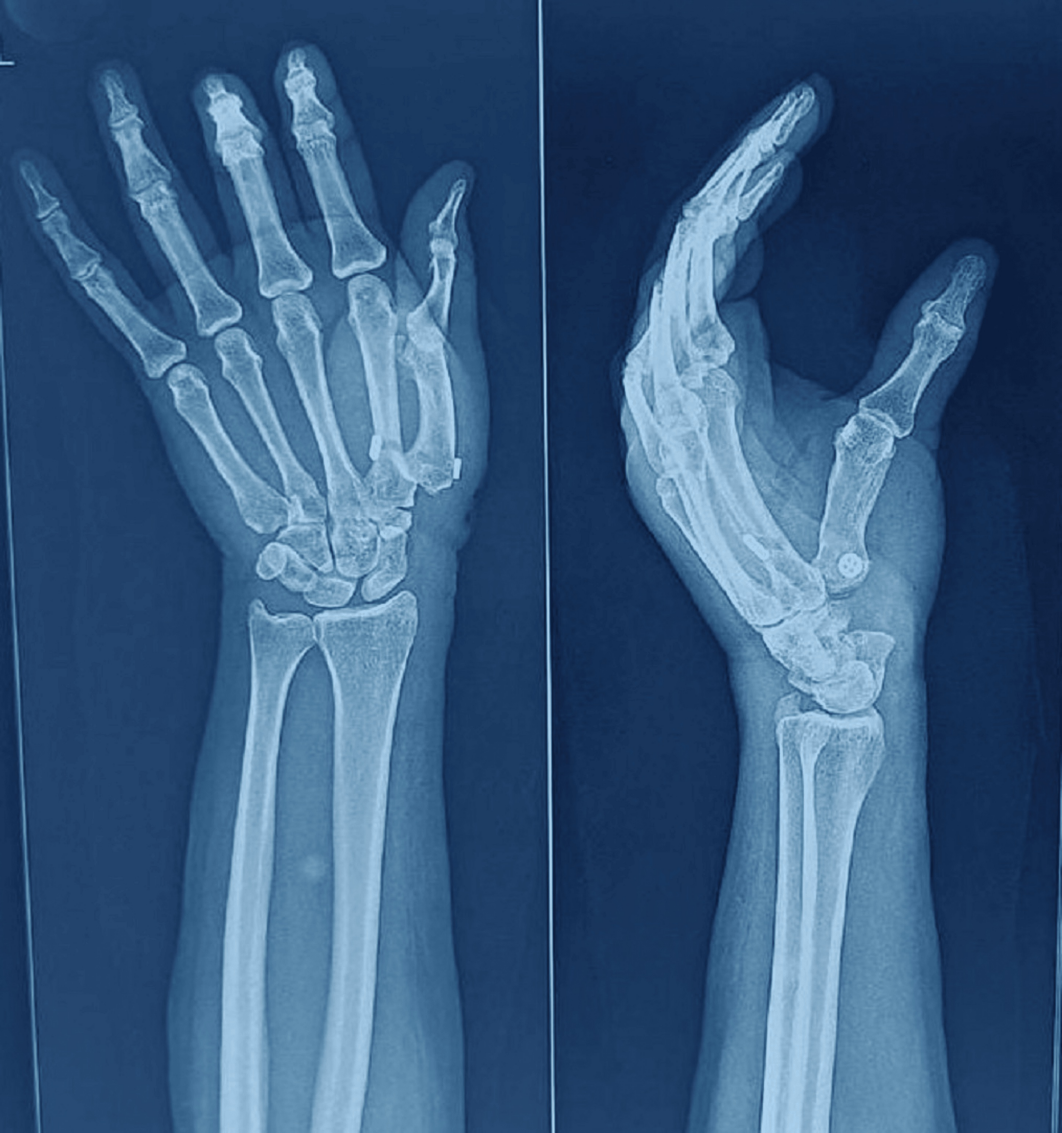
Postoperative Radiographs

Typically a thumb spica splint was given to protect the surgical site and promote healing. Early mobilization and range of motion exercises guided by a hand therapist to prevent stiffness and promote functional recovery was started. Regular monitoring of wound healing, pain levels, grip strength, and range of motion during postoperative visits was done and radiographs (Figure 3) were taken. Outcome assessment was done using Visual Analog Scale (VAS) to quantify pain levels. Functional assessment was done by measurement of grip strength and evaluation of thumb range of movement. Patient-reported outcomes were used by utilization of standardized questionnaires i.e. QuickDASH and Nelson score to assess functional outcomes and patient satisfaction.

## Results

In our study of 20 patients who underwent trapeziectomy combined with Mini TightRope suspensionplasty for first CMC joint arthritis, we observed significant improvements across several key metrics. Preoperatively, patients reported an average pain score of 8.3 on a 0-10 VAS. Postoperatively, pain scores decreased significantly to an average of 1.5 at six months follow-up (Table 2). All patients experienced a marked reduction in pain intensity, indicating effective pain management following surgery. Grip strength measurements showed substantial improvement postoperatively. On average, grip strength increased by 40% compared to preoperative levels. This improvement suggests enhanced thumb function and strength for daily activities. Range of motion assessments demonstrated notable gains in thumb mobility. Approximately 85% of patients achieved near-normal range of motion in the thumb joint. Improved joint flexibility contributes to enhanced functionality and performance of daily tasks.

**Table 2:** Pre and Postoperative VAS Score The table represents pre- and postoperative Visual Analogue Score (VAS) with P value of <0.05 considered significant.

Serial number	Sex	VAS score		P value
		Pre op	Post op	0.014
1	Male	8	2	
2	Female	9	1	
3	Female	7	1	
4	Female	8	2	
5	Female	9	1	
6	Male	8	2	
7	Male	7	1	
8	Female	9	1	
9	Female	8	2	
10	Female	7	1	
11	Female	9	2	
12	Male	8	1	
13	Female	7	1	
14	Female	9	2	
15	Female	8	1	
16	Female	7	2	
17	Male	9	1	
18	Female	8	2	
19	Female	7	1	
20	Female	9	1	
Average		8.3	1.5	

Patient-reported outcome measures, including the Disabilities of the Arm, Shoulder, and Hand (DASH) questionnaire [6], indicated high levels of satisfaction (Table 3). Ninety percent of patients reported improved ability to perform activities of daily living without significant limitations. Patients expressed overall satisfaction with the surgical outcome and reported a positive impact on their quality of life.

**Table 3:** Pre- and Postoperative QuickDash Score QuickDash - Quick Disabilities of the Arm, Shoulder, and Hand, P-value <0.05 considered significant

Serial number	Sex	QuickDASH Score		P value
		Pre op	Post op	0.024
1	Male	60	18	
2	Female	62	16	
3	Female	58	15	
4	Female	59	20	
5	Female	61	14	
6	Male	60	19	
7	Male	58	13	
8	Female	62	17	
9	Female	59	21	
10	Female	58	12	
11	Female	62	22	
12	Male	60	11	
13	Female	58	14	
14	Female	61	23	
15	Female	60	10	
16	Female	58	16	
17	Male	62	19	
18	Female	59	24	
19	Female	58	11	
20	Female	61	15	
Average		59.4	15.4	

Another self-administered questionnaire, the Nelson score [7], was used to assess the outcome following surgery which showed an average of 23.1 (Table 4) and combined with the physical findings proved external validity for the study.

**Table 4:** Postoperative Nelson Score Data calculated as N and Mean of the values calculated.

Serial number	Sex	Post operative nelson score
1	Male	25
2	Female	22
3	Female	28
4	Female	20
5	Female	26
6	Male	24
7	Male	18
8	Female	23
9	Female	27
10	Female	19
11	Female	21
12	Male	29
13	Female	17
14	Female	25
15	Female	22
16	Female	16
17	Male	30
18	Female	26
19	Female	24
20	Female	18
Average		23.1

There were no major complications such as infection, nerve injury, or implant failure observed in our cohort. Minor complications included transient stiffness and mild discomfort, which resolved with conservative management. The post operative video (Video 2) shows good functional outcome and range of motion.

**Video 2:** Post Operative Clinical Video

## Discussion

Our aim was to determine the efficacy of the combined approach, trapeziectomy followed by suspensionplasty using Mini TightRope. Our results demonstrate significant improvements in pain relief, grip strength, and range of motion following trapeziectomy with Mini TightRope suspensioplasty. The TightRope has been widely used in other areas of the body and is widely used by hand surgeons now. It consists of polyethylene wire passing between two buttons. It is a sustitute to traditional K-wire which was earlier used, with the added benefit of early return to mobilization. Our study has demonstrated a short-term follow-up with significant improvement in the functional variables. The substantial reduction in pain scores from an average of 8.3 preoperatively to 1.5 postoperatively highlights the efficacy of this combined surgical approach in managing symptomatic thumb arthritis. The improvement in grip strength by 40% and achievement of near-normal range of motion in 85% of patients further support the functional benefits of stabilizing the first CMC joint while addressing structural deformity through trapeziectomy. Yao and Song found similar results in their study [8]. Also in literature, for trapeziectomy, the reported DASH scores for long-term follow-up are higher than those for the primary group after suspensionplasty [9,10]. Furthermore, De Smet et al. [11] found a significant correlation between the DASH scores and the functional and subjective outcomes for trapeziectomy with and without a ligament reconstruction and tendon interposition (LRTI), which is in line with our results. One of the primary concerns with traditional trapeziectomy alone is the potential loss of thumb stability and strength. The addition of Mini TightRope suspensionplasty aims to mitigate this risk by providing dynamic stabilization of the thumb metacarpal, thereby preserving joint mechanics and optimizing functional outcomes. Our findings suggest that this approach effectively balances pain relief with maintenance of thumb mobility, essential for performing daily activities and maintaining quality of life. Patient-reported outcomes play a crucial role in evaluating surgical success and patient satisfaction. The high satisfaction rates observed in our study, with 90% of patients reporting improved ability to perform activities of daily living, underscore the positive impact of surgery on overall quality of life. These outcomes are consistent with previous studies highlighting the importance of addressing both pain management and functional restoration in the treatment of thumb arthritis [12,13]. Another study by Cox et al. [4] also described the technique following a hemi-trapeziectomy and detailed procedure of implant postioning and use of flouroscopy. In our cohort, we followed the same method of tensioning and intraoperative ballotment test under flouroscopy for better results. The post operative thumb opposition, pain and functional outcomes were favourable in our study. An easy recognizable benefit from this new technique is no need of harvesting any tendons and thereby reducing the morbidity and operative time.

While our study provides promising results, several limitations warrant consideration. Relatively small sample size (20 patients) may limit the generalizability of findings. Variability in patient demographics, severity of arthritis, and individual healing responses could influence outcomes. Additionally, longer-term follow-up beyond six months is essential to assess the durability of pain relief, joint stability, and long-term functional outcomes. Despite all this, our study provides the short term results with this novel technique with quick post operative recovery and avoids the need of harvesting any grafts. Future research should focus on prospective, randomized controlled trials with larger cohorts to further validate the efficacy and durability of trapeziectomy with Mini TightRope suspensionplasty. Comparative studies evaluating this approach against other surgical techniques, such as arthroplasty or arthroscopic procedures, could provide insights into optimal treatment algorithms for thumb arthritis. Furthermore, investigating biomechanical aspects and long-term implant performance will contribute to refining surgical techniques and enhancing patient outcomes.

## Conclusions

Our study demonstrates favourable outcomes for trapeziectomy with Mini Tightrope suspensionplasty in managing first CMC joint arthritis. The procedure effectively alleviates pain, improves grip strength, enhances range of motion, and enhances overall patient satisfaction. These findings support the use of this combined surgical approach as a viable option for patients seeking relief from thumb arthritis, highlighting its potential to optimize functional outcomes and improve quality of life. Further research with larger sample sizes and long-term follow-up is recommended to validate these results and refine treatment protocols for enhanced patient care.

## References

[1] Jager T (2021). Total trapeziectomy. Hand Surg Rehabil.

[2] Vermeulen GM, Slijper H, Feitz R, Hovius SE, Moojen TM, Selles RW (2011). Surgical management of primary thumb carpometacarpal osteoarthritis: a systematic review. J Hand Surg Am.

[3] Wolfe T, Chu JY, Woods T, Lubahn JD (2014). A systematic review of postoperative hand therapy management of basal joint arthritis. Clin Orthop Relat Res.

[4] Cox CA, Zlotolow DA, Yao J (2010). Suture button suspensionplasty after arthroscopic hemitrapeziectomy for treatment of thumb carpometacarpal arthritis. Arthroscopy.

[5] Yao J, Song Y (2013). Suture-button suspensionplasty for thumb carpometacarpal arthritis: a minimum 2-year follow-up. J Hand Surg Am.

[6] Hosokawa T, Tajika T, Suto M, Chikuda H (2020). The Quick Disabilities of the Arm, Shoulder, and Hand (QuickDASH) scores in 961 Japanese volunteers. J Orthop Surg (Hong Kong).

[7] Citron N, Hulme CE, Wardle N (2007). A self-administered questionnaire for basal osteoarthritis of the thumb. J Hand Surg Eur Vol.

[8] Jørgensen RW, Anderson KA, Jensen CH (2023). Mini tightrope suspension allows for accelerated rehabilitation following ligament reconstruction interposition arthroplasty of the basal joint of the thumb. J Hand Microsurg.

[9] De Smet LS, Sioen W (2007). Basal joint osteoarthritis of the thumb: trapeziectomy, with or without tendon interposition, or total joint arthroplasty? A prospective study. Eur J Orthop Surg Traumatol.

[10] Saab M, Chick G (2021). Trapeziectomy for trapeziometacarpal osteoarthritis. Bone Jt Open.

[11] De Smet L, Sioen W, Spaepen D, van Ransbeeck H (2004). Treatment of basal joint arthritis of the thumb: trapeziectomy with or without tendon interposition/ligament reconstruction. Hand Surg.

[12] van Laarhoven CM, Treu S, Claasen LC, Van Heijl M, Coert JH, Schuurman AH (2022). Trapeziectomy and alternative suspension technique in thumb carpometacarpal arthritis: patient-reported outcome measures. J Hand Surg Glob Online.

[13] Leclercq C (2015). Thumb CMCJ arthritis: a new technique of suspensionplasty (Mini tightrope). BMC Proc.

